# Acute cerebral infarction with acute myocardial infarction due to patent foramen ovale: A case report

**DOI:** 10.1097/MD.0000000000020054

**Published:** 2020-05-08

**Authors:** Jinghong Chen, Rui Li, Jingjing Chen, Jingru Zhao, Na Li, Sujuan Sun, Baoming Yang

**Affiliations:** a Department of Neurology; b Department of Ultrasound, Hebei General Hospital; c Department of Hepatobiliary Surgery, the Fourth Hospital of Hebei Medical University, Shijiazhuang, P. R. China.

**Keywords:** cerebral infarction, embolization., myocardial infarction, paradoxical embolism, patent foramen ovale

## Abstract

**Rationale:**

Patent foramen ovale (PFO) is not considered to be the main cause of stroke and is classified as the infarction of undetermined cause. The relationship between PFO and cerebral embolism is still unclear and cerebral embolism accompanied with coronary artery embolization in PFO patient is rare. In this case, we reported a patient with PFO suffered acute cerebral and myocardial infarction simultaneously, and analyzed the source of emboli and potential pathogenesis.

**Patient concerns:**

A 53-year-old female presented with chief complaints of intermittent palpitations and chest tightness for 6 years, aggravated for 3 days.

**Diagnoses:**

During the hospitalization, acute cerebral infarction and acute myocardial infarction occurred at the same time in the patient. The patient felt paroxysmal abdominal pain repeatedly. Finally, we detected PFO in the patient

**Interventions:**

Double antiplatelet therapy was given to the patient of acute cerebral and myocardial infarction with PFO.

**Outcomes:**

Two weeks after the onset of the disease, the condition was relatively stable. But after 2 months, the patient experienced repeated heart failure, transthoracic echocardiography manifested no significant change in the PFO gap but significant cardiac function reduction.

**Lessons:**

Although a growing number of people are aware that PFO is a risk factor for arterial embolization especially when coexisting with atrial septal aneurysm, a significant proportion of patients have paradoxical embolism after PFO closure. Therefore, transesophageal echocardiography should be routinely performed to find the possible cause of embolism when infarction of undetermined cause occurs, and PFO closure and anti-platelet aggregation or anticoagulant therapy should be given at the same time in order to reduce the occurrence of arterial thrombosis.

## 1 Introduction

Cardiovascular disease and stroke produce immense health and economic burdens globally.^[1]^ A growing body of evidence has suggested that atherosclerosis is still a critical risk factor for ischemic stroke in different countries, genders, and lifestyles.^[2]^ Infarction of undetermined cause (IUC) accounts for 35% to 40% of ischemic stroke.^[3]^ Patent foramen ovale (PFO) is not considered to be the main cause of stroke classified as the category of IUC,^[4]^ and acute myocardial infarction caused by embolism is rarely reported. The case we reported is a patient with acute cerebral and myocardial infarction, and we will analyze the source of emboli and its pathogenesis in detail.

## 2 Case presentation

**Figure 1:**
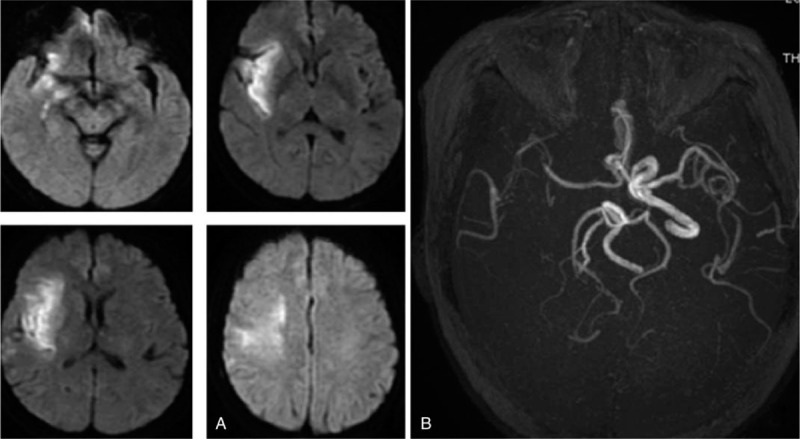
Imaging studies. (A&B) Cranial magnetic resonance imaging in patient showed acute multiple cerebral infarction, most of the right internal carotid artery not shown exactly; the right middle cerebral artery imaging shallow.

A 53-year-old female presented with chief complaints of intermittent palpitations and chest tightness for 6 years, aggravated for 3 days. Six years ago, the patient got palpitation, chest tightness, and pulse acceleration (self-measured and the specific data was not clear) with no obvious causes. Five years ago, the patient had irregular uterine bleeding. When the hemorrhagic amount increased, the patient was prone to palpitations and chest tightness. She visited a local hospital for treatment and the coronary angiography showed no abnormalities. Three days prior to the admission, the symptoms of palpitations and chest tightness aggravated after activities, and nocturnal paroxysmal dyspnea appeared, accompanied by intermittent abdominal pain. Thus, she went to the local hospital again, electrocardiogram showed III degree atrioventricular block. The local diagnosis was coronary atherosclerotic heart disease, arrhythmia, and she received appropriate treatment, however, the symptoms did not improve significantly. Therefore, on the fourth day, she visited outpatient clinics and was admitted to the department of cardiology with the diagnosis of coronary atherosclerotic heart disease in our hospital. Physical examination revealed the body temperature of 35.7°C, heart rate of 78 beats per minute, respiratory rate of 18 breaths per minute, and blood pressure of 114/80 mm Hg. The patient was alert and cooperative. There was no cyanosis on the lips and no dilatation of bilateral jugular vein. Moist rales, without dry rales, can be heard in the left lung. The heart rate was 78 beats per minute with regular rhythm and no murmur was heard in the area of each valve auscultation. The abdomen was soft without tenderness or rebound tenderness. Liver and spleen were not palpable. Mobile dullness was negative. There was no lower extremity edema. Preliminary diagnosis was arrhythmia, III degree atrioventricular block, heart failure, irregular uterine bleeding cause to be investigated. After admission, the electrocardiogram demonstrated that sinus rhythm and I degree atrioventricular block. Cardiac color doppler ultrasound showed dilatated left atrium and right heart, a small amount of mitral valve regurgitation, a large amount of tricuspid valve regurgitation, and the increased pulmonary artery pressure with a small amount of pericardial effusion. Chest radiography showed increased heart shadow and a small amount of pleural effusion on the left. On the day of admission, the patient appeared restlessness with eyes gazing to the right, and felt weakened in the left limbs. Cranial computed tomography (CT) showed no abnormalities. The abdominal CT showed a small amount of pleural, peritoneal and pericardial effusion. Then the patient was transferred to the department of neurology. Neurologic examination: the patient was sleepy and could answer simple questions correctly. The eyes were fixed to the right. Pupils were 4 mm and briskly reactive to light. The forehead wrinkles and nasolabial folds were symmetric with normal eye closure. Tongue was middle with normal movement. The left upper limb muscle strength was grade 1, and the left lower limb was grade 3. Muscle tone was increased in the left limb. Motor examination of the right limb were normal. Bilateral tendon reflexes were symmetrical. Deep and superficial sensation were intact. Babinski sign was positive on the left side. Meningeal irritation sign was negative. Cranial magnetic resonance imaging (MRI) showed that right frontotemporal, right insula, bilateral basal ganglia, and left parietal lobe were presented with acute multiple cerebral infarction. Right internal carotid artery was not clear shown, and the right internal carotid artery and middle cerebral artery were not shown clearly (Figs. 1A and 1B). D-dimer was 3292 g/L. The level of troponin was 2.58 g/mL. The electrocardiogram was considered as an acute myocardial infarction. Blood routine examination showed white blood cell count was 12.53 x 10^9^/L and neutrophil ratio was 91.3%. Biochemistry showed total protein of 57.2 g/L, albumin of 29.6 g/L, aspartate aminotransferase of 95 U/L, lactate dehydrogenase of 703 U/L, hydroxybutyrate dehydrogenase of 708 U/L, sodium of 133.0 mmol/L, and creatine kinase of 190 U/L. Thyroid stimulating hormone level was 4.400 U/mL. Carbohydrate antigen 125 was elevated slightly. Review of myocardial enzyme showed elevated aspartate aminotransferase (155 U/L), lactate dehydrogenase (1385 U/L), and hydroxybutyrate dehydrogenase (1355U/L). Brain natriuretic peptide was 10733.00 g/mL. The level of troponin and D-dimer was also elevated to 2.12 g/mL and 747 μg/L separately. Diagnosis showed acute cerebral infarction, acute myocardial infarction, heart insufficiency, heart failure. We gave the patient atorvastatin, cilostazol, edaravone, oxiracetam, butylphthalide, compound dextran in order to dilate vascular, nurture nerves, clear free radical, and improve microcirculation.

The patient felt repeated abdominal pain with no positive abdominal sign 1 week later. We considered the possible cause of mesenteric artery embolization, however, the abdominal pain relieved after 3 days. The left lung could be heard moist rales in the next week. The patient could follow the commands and her speech was clear and fluent. Both eyes were able to move coordinately in all directions. The left muscle strength increased to grade 3 of upper limb, and to grade 3+ of lower limb. We reviewed the laboratory tests 10 days later. The troponin was significantly lower than before (0.105 g/mL); myocardial enzyme also decreased, including aspartate aminotransferase (89 U/L), lactate dehydrogenase (374 U/L), hydroxybutyrate dehydrogenase (335 U/L), creatine kinase (24 U/L). Blood routine examination showed that neutrophil ratio was 85.7%; brain natriuretic peptide was 2678 pg/mL.

**Figure 2:**
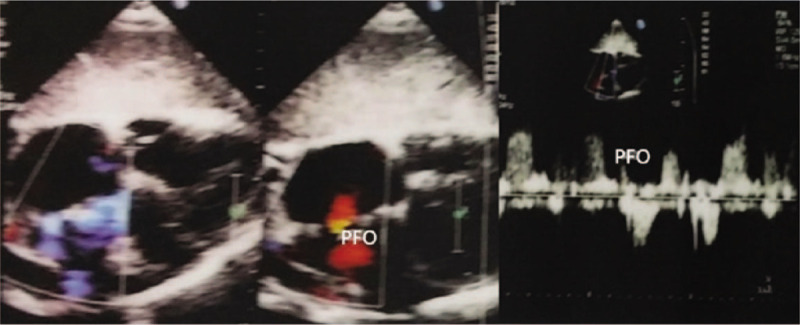
Echocardiography revealed patent foramen ovale, left atrium and right heart dilatated, a large number of tricuspid valve regurgitated.

The patientʼs condition was stable than before. In order to search the cause of the disease, we checked cardiac color ultrasound again and found PFO (Fig. 2). The dynamic electrocardiogram was used to evaluate the degree of atrioventricular block. Combined with troponin, myocardial enzyme, and electrocardiogram, we diagnosed acute myocardial infarction. Combined with physical examination and MRI, acute cerebral infarction was also clearly diagnosed. Discharge diagnosis was acute cerebral infarction, acute myocardial infarction, cardiac insufficiency, heart failure, arrhythmia with I degree atrioventricular block, and PFO. The study protocol was approved by the Ethics Committee of the Hebei General Hospital and the patient gave consent for publication of this case report.

## 3 Discussion

The etiology classification of cerebral infarction mainly referred to TOAST classification which was classified into 5 categories and proposed by American Adams in 1993, namely large artery atherosclerosis, cardiogenic embolization, small artery occlusion, other clear etiology, and IUC. PFO is classified as IUC. Although PFO accounted for 25% of the normal adults, the relationship between PFO and cerebral embolism is still unclear and cerebral embolism accompanied with coronary artery embolization in PFO patient is rare.

Fetal lung is in a state of tension and does not need to bear respiratory function. High resistance to pulmonary circulation and blood flow is very small, so the blood into the right atrium must be able to enter the left atrium through the atrial septum in order to adapt to the special cycle of fetal physiological requirements. In growth and development process, the atrial septum has been the heart of the hole. PFO is the heart atrial septal embryonic period of a physiological channel. After the birth of 5 to 7 months, atrial septum secondary septum and the primary septum begin to adhere and fuse to form a permanent atrial septum in most people, if not, PFO appears. Autopsy studies have confirmed that the incidence of PFO in adults is between 17% to 35%.^[5]^ Right-to-left shunting (RLS) occurs when the right atrial pressure increases such as the end of diastole, the start of systole, coughing, laughing, sneezing, and Valsalva action. The venous system and right atrium thrombus through the heart of the traffic from the right heart into the left ventricular system caused by embolism is known as paradoxical embolism (PDE). In 1972, Meiste proposed the diagnostic criteria for PDE, the formation of which should have the following 4 conditions:

(1) no artery embolization associated with left ventricular system or large artery embolism;
(2) presence of deep vein thrombosis with or without pulmonary embolism;
(3) RLS, such as internal or external abnormal traffic in the heart or systemic circulation and pulmonary circulation venous fistula; right heart system pressure increased, persistent (such as pulmonary hypertension) or transient (such as Valsalva action or cough).^[6]^ In PFO patient, embolic origin may have the following 3 kinds:
  - venous thrombosis is a major source of PDE in patients with PFO;
  - (2) some of small thrombus, which could not be detected by the current imaging techniques, may come from the varicose veins or small hemorrhoids;
  - very small pressure difference between the heart chamber coupled with uneven surface oval pits which can lead to blood stasis and the formation of PFO thrombosis in situ.
  - Therefore, embolization can also be derived from the PFO itself. Since PFO patients are at risk of PDE in certain circumstances, which kind of PFO patients are more prone to develop PDE? Despite the notion that stroke is caused by PDE of PFO,^[7]^ PFO associated with occult strokes is still unclear. At present, it is believed that the size of PFO and the combination of atrial septal aneurysm are closely related to PDE. Generally, the larger the PFO is and the greater the flow rate is, the higher the incidence of PDE will be. The risk of embolism is significantly higher in patients with PFO combined with atrial septal aneurysm.^[8,9]^ The incidence of PDE accounted for 2% to 16% of arterial embolization. It is found that the primary atrial septal activity was related to stroke, activity > 6.5 mm or resting PFO-RLS was at high risk of the occurrence and recurrence of stroke.^[10]^ It had been shown that PFO was an independent risk factor for cerebrovascular events especially in young adults and unexplained stroke. PDE embolism from the venous system mainly occurred in the brain, followed by the limbs and internal organs less involved in coronary artery. It was reported that the incidence of myocardial infarction was 1% to 7%, with coronary artery normal. PFO could be used as a venous embolus to reach the coronary artery channel. In addition, PFO could also act as a vasoactive substance in the coronary channel, resulting in coronary vasospasm. A total of 416 patients diagnosed with PFO were followed up for 8 years. In this study, we found that there were 219 cases of stroke caused by unknown reasons, in which there were 38 cases of migraine, transient ischemic attack (TIA) occurred in 80 patients, and 12 cases (2.9%) were diagnosed as systemic embolism. A total of 8 patients had elevated cardiac biomarkers, electrocardiogram changes and abnormal left ventricular wall motion. There was no evidence of coronary angiography of coronary artery occlusion to show myocardial infarction in the diagnosis of acute myocardial infarction. A total of 4 patients underwent systemic arterial embolization with evidence in peripheral including the popliteal artery, ophthalmic artery and the brachial artery. Although most of the contradictory embolism travelled to the brain, other embolism was also associated with PFO. In addition to thromboembolism, there might be contradictory vasoactive chemicals that might induce a strong coronary artery spasm, leading to the myocardial infarction. Diagnosis was usually challenging due to the lack of clear criteria and ruling out other possible causes.^[11]^

PFO was mainly diagnosed by echocardiographic diagnosis including transthoracic echocardiography (TTE), transesophageal echocardiography, and contrast transcranial Doppler or even CT and MRI.^[12]^ The PFO suspected by TTE should check contrast transthoracic echocardiography examination and the sensitivity of contrast transthoracic echocardiography could reach 63% to 100%. It was a safe and effective treatment of PFO closure in the prevention of recurrent stroke in multiple clinical studies.^[13,14]^ Meta-analysis found that after percutaneous PFO closure, stroke recurrence rate was 0.47% and TIA was 0.85% in 3819 patients.^[15]^ Wahl et al compared the results of PFO closure and anticoagulation or antiplatelet therapy in the treatment of 308 cases of patients with follow-up for up to 10 years, and found that closure could significantly reduce the mortality and morbidity of stroke and TIA.^[16]^ Residual and new RLS were still observed in 120 patients treated with closure for more than 5 years by contrast transcranial Doppler.^[17]^ Another 730 cases of patients with PFO following up of 6 years showed device closure could be a good and safe long-term solution to atrial shunt. Recurrent neurological damage might reflect that additional common disease risk factors had nothing to do with potential PDE, but in relationship with other cerebrovascular diseases risk factors such as hypertension, arteriosclerosis.^[18]^

Drug therapy aims to prevent recurrence of stroke or TIA in patients with PFO. It is not sure that aspirin or warfarin is better. The PFO in Cryptogenic Stroke Study showed that 2-year stroke recurrence rate was 13.2% in PFO patients with stroke taking aspirin group, 16.5% in warfarin treatment group, and no significant difference between the 2 groups, but warfarin significantly increased the risk of small bleeding.^[19]^ Therefore, antiplatelet therapy (aspirin 3 to 5 mg / (kg·d) or clopidogrel (75 mg/d)) is the preferred treatment. We can use warfarin when there is still the recurrence of stroke, or combination of deep vein thrombosis and hypercoagulable state in antiplatelet therapy. At present, there is a lack of relevant data or experience in the use of new oral anticoagulants. Drug treatment also has many disadvantages, such as the need for long-term treatment, bleeding, the poor compliance, and so on. Hence, PFO is not only a potential risk and changeable factor for stroke, but also a risk factor for stroke recurrence.

In this case, the patient underwent the heart of color Doppler ultrasound more than once, and no PFO was found. We can see that TTE detection rate is low, partly attributed to the examiner technology. In the local hospital coronary angiography, there was no visible vascular abnormalities. The acute myocardial infarction might not be due to coronary artery itself, and embolus derived from other parts should be considered. The acute cerebral infarction and acute myocardial infarction occurred at the same time, and the patient had abdominal pain. Thus, we considered it was the mesenteric artery embolization. We considered the embolism was from the heart, with a monism to explain. Finally, we found PFO through repeated echocardiography examination. It showed bidirectional blood clearly, embolism might be the PFO thrombosis in situ or reverse thrombosis originating from venous thrombosis. As a result of severe condition, large area of cerebral infarction and acute myocardial infarction, poor heart function combined with economic difficulties, the patient did not choose the PFO closure, but choose the double antiplatelet therapy. Two weeks after the onset, the condition was relatively stable. But after 2 months, the patient had repeated heart failure, TTE manifested no significant change in PFO gap but significantly cardiac function reduction. There might appear re-embolism at any time, thus the patient was suggested to continue the double antiplatelet therapy.

## 4 Conclusions

Although a growing number of people are aware that PFO is a risk factor for arterial embolization especially when coexisting with atrial septal aneurysm, a significant proportion of patients have PDE after oval foramen closure. Therefore, transesophageal echocardiography should be routinely checked as far as possible to find the cause of embolism early when IUC appears, and closure of foramen ovale, anti-platelet aggregation or anticoagulant therapy should be used to reduce the occurrence of arterial thrombosis.

## Author contributions

**Conceptualization:** Jinghong Chen, Jingjing Chen, Jingru Zhao, Na Li, Sujuan Sun.

**Supervision:** Baoming Yang.

**Writing – original draft:** Rui Li.

**Writing – review & editing:** Jingru Zhao, Baoming Yang.
